# Soluble CD137: A Potential Prognostic Biomarker in Critically Ill Patients

**DOI:** 10.3390/ijms242417518

**Published:** 2023-12-15

**Authors:** Ulrich Räth, Patricia Mester, Herbert Schwarz, Stephan Schmid, Martina Müller, Christa Buechler, Vlad Pavel

**Affiliations:** 1 Department of Internal Medicine I, Gastroenterology, Hepatology, Endocrinology, Rheumatology, and Infectious Diseases, University Hospital Regensburg, 93053 Regensburg, Germany; ulrich.raeth@stud.uni-regensburg.de (U.R.); patricia.mester@klinik.uni-regensburg.de (P.M.); stephan.schmid@klinik.uni-regensburg.de (S.S.); martina.mueller-schilling@klinik.uni-regensburg.de (M.M.); vlad.pavel@klinik.uni-regensburg.de (V.P.); 2 Department of Physiology, and Immunology Programme, Yong Loo Lin School of Medicine, National University of Singapore, MD9, Singapore 117597, Singapore; phssh@nus.edu.sg

**Keywords:** sCD137, COVID-19, sepsis, bacterial infection, survival

## Abstract

T cell depletion and functional impairment are characteristics of sepsis. CD137 is a costimulatory receptor on activated T cells, while soluble CD137 (sCD137) inhibits CD137 signaling. This study found elevated sCD137 levels in the plasma of patients with systemic inflammatory response syndrome (SIRS), sepsis, or septic shock compared to healthy controls. The sCD137 levels negatively correlated with the C-reactive protein and positively with procalcitonin and interleukin-6. There was no difference in sCD137 levels based on ventilation, dialysis, or vasopressor treatment. Patients with SARS-CoV-2, Gram-positive, or Gram-negative bacterial infections had similar sCD137 levels as noninfected individuals. Notably, higher plasma sCD137 levels were observed in non-survivors compared to survivors in both the SIRS/sepsis group and the SARS-CoV-2 subgroup. In conclusion, plasma sCD137 levels are associated with severe illness and survival in critically ill patients.

## 1. Introduction

Sepsis is a complex disease characterized by a multifaceted immune response [1,2]. In septic patients, the initial hyperinflammatory phase transitions to a hypoinflammatory state. Understanding the suppressed immune cell function in this later stage is key to addressing the challenges associated with sepsis mortality [3]. Notably, while lymphopenia is observed in about 50% of septic patients, ongoing research is shedding light on its relation to disease severity and outcome [4].

Activated T cells express the costimulatory molecule CD137, and the binding of its ligand CD137L induces T cell proliferation and inhibits T cell apoptosis [5,6,7]. CD137 is also expressed by natural killer cells, B cells, granulocytes, and monocytes/macrophages [8,9]. Agonistic anti-CD137 antibodies cause the polarization of macrophages to an M1 type. These macrophages have a high phagocytic activity and produce inflammatory cytokines [8]. CD137 signaling is also required for the early activation of neutrophils in bacterial infections [10].

CD137-null mice infected by the Gram-positive bacteria *Listeria monocytogenes* had more severe disease and less survival than the wild-type controls. The neutrophils of these animals showed an impaired phagocytic activity and an inappropriate production of reactive oxygen species [10]. The survival of the CD137-null mice was, however, improved upon simultaneous infection with Gram-negative and Gram-positive bacteria [11]. Lipopolysaccharide injection caused the death of the wild-type controls but elicited a less-pronounced cytokine storm in the CD137-deficient mice, which all survived [12]. Whether the CD137-null mice infected with Gram-negative bacteria have a survival advantage has not been described so far. Recombinant CD137-Fc improved the recovery of mice from Gram-negative *Acinetobacter pneumonia* in the immunosuppressive phase of sepsis, suggesting a protective role of CD137 in Gram-negative infection and the hypoinflammatory state [13].

CD137 activity is modulated not just by its ligand CD137L, but also by a soluble variant, soluble CD137 (sCD137) [5]. Produced either by the differential splicing of CD137 or by shedding via a disintegrin and metalloproteinases (ADAMs) 10 and 17, sCD137 lacks the transmembrane domain [14,15,16]. This soluble form can interfere with CD137 binding to CD137L, likely inhibiting T cell costimulation [5] and increasing activation-induced lymphocyte death [14]. Furthermore, sCD137 has shown potential in reducing CD4 T cell activation even without CD137L-expressing cells, suggesting its capability to modulate T cell activity via multiple routes [17]. Excitingly, recombinant sCD137 has been found to temper both Th1 and Th2 polarization, as well as reduce key inflammatory mediators, like tumor necrosis factor, interferon gamma, and interleukin 13, in cell media [18]. Moreover, sCD137 may inhibit the release of proinflammatory cytokines, such as interleukin-6 (IL-6) and tumor necrosis factor, by activated monocytes and B cells [5,9].

The differentiation of CD4 T cells to Th1, Th2, and Th17 cells is impaired in sepsis. T cell exhaustion after prolonged exposure to inflammatory stimuli is associated with an increased expression of inhibitory receptors, such as programmed cell death (PD) ligand 1, which dampens the immune response and lowers the proliferation of PD-1-expressing immune cells [19,20]. In view of the immune-suppressive effects of sCD137, it seems likely that this molecule also contributes to impaired immunity in sepsis. We are, however, only aware of one study having analyzed circulating levels of sCD137 in severely ill patients. This investigation was performed in patients with severe acute respiratory syndrome coronavirus type 2 (SARS-CoV-2) infection, which can cause viral sepsis [21,22]. Serum sCD137 levels in patients with severe COVID-19 were higher in contrast to healthy controls, and were related to disease severity [22].

The serum sCD137 of healthy donors are relatively low, and a pronounced upregulation occurred in the sera of patients with chronic lymphocytic leukemia, autoimmune diseases, or pancreatitis [5,23,24]. The serum sCD137 level of patients with hepatitis C virus and alcohol-related liver cirrhosis also increased [18]. Elevated levels of immune-cell-activity-inhibitory molecules may contribute to higher infection rates of patients with chronic liver diseases [25]. Accordingly, it has been shown that the blockage of PD ligand 1, which is highly expressed in the liver macrophages of patients with chronic liver diseases, increased the antimicrobial activities of these phagocytes [26].

Our study aimed to uncover the associations between plasma sCD137 and the causes of severe illness, as well as disease outcomes. By understanding these links in a cohort of patients with SIRS, sepsis, or septic shock, we hope to contribute valuable insights for the development of novel biomarkers and to enhance patient care.

## 2. Results

### 2.1. Plasma sCD137 of SIRS/Sepsis Patients

Table 1 describes the whole cohort of 153 patients with SIRS/sepsis. The median age of the patients was 59 years, with approximately a third being women. This cohort included 23 patients who had sepsis because of SARS-CoV-2 infection. The COVID-19 subgroup did not differ from the whole cohort with regard to sex, age, and the measured laboratory parameters. The whole cohort and the COVID-19 patients had a similar age and sex distribution in comparison to the healthy controls. The patient cohorts, i.e., the whole cohort and the subgroup of COVID-19 patients, had higher plasma IL-6 levels than the controls (Table 1). Thirty of the one hundred and fifty-three patients had liver cirrhosis. Patients with liver cirrhosis had a lower CRP in comparison to the whole cohort of SIRS/sepsis patients, whereas all other parameters were similar (Table 1).

**Figure 1:**
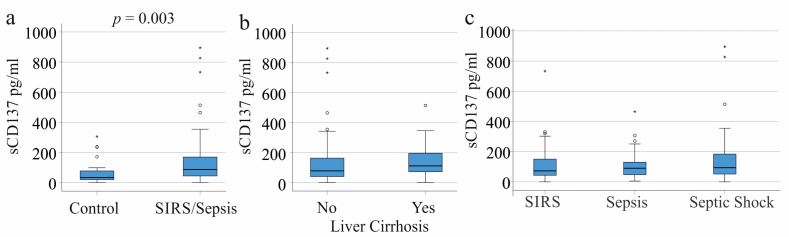
The sCD137 in the plasma of the controls and the SIRS/sepsis patients. (**a**) Plasma sCD137 levels of the controls and the SIRS/sepsis patients; (**b**) Plasma sCD137 levels of the SIRS/sepsis patients with and without liver cirrhosis; (**c**) Plasma sCD137 levels of the SIRS/sepsis patients with SIRS, sepsis, and septic shock. Small circles and asterisks mark outliers.

The plasma sCD137 levels in the 153 SIRS/sepsis patients were higher than in the 22 age- and sex-matched controls (*p* = 0.003; Figure 1a). The receiver operating characteristic (ROC) curve for the diagnosis of sepsis showed an area under the curve (AUC) of 0.697. The Youden index determined a cut-off value of 49.43 pg/mL sCD137 for the diagnosis of sepsis, with a sensitivity of 72% and a specificity of 73%.

No significant gender differences in the plasma sCD137 levels were found in both the control (*p* = 0.606) and patient groups (*p* = 0.882). There was no correlation between age and sCD137 levels among the patients, with a Spearman correlation coefficient r = −0.040 (*p* = 0.627). However, the control cohort showed a positive correlation between sCD137 levels and age (r = 0.431, *p* = 0.045).

Previous studies indicated higher serum sCD137 levels in patients with liver cirrhosis [18]. The plasma sCD137 levels of the 30 SIRS/sepsis patients with liver cirrhosis tended to be elevated, though not significantly (*p* = 0.053; Figure 1b). As liver cirrhosis did not significantly alter the plasma sCD137 levels in the sepsis cohort, these patients were not excluded from further analysis.

The plasma sCD137 levels were consistent across the patients with SIRS, sepsis, and septic shock (Figure 1c).

### 2.2. Plasma sCD137 Levels of the SIRS/Sepsis Patients in Relation to Underlying Diseases and SARS-CoV-2 Infection

**Figure 2:**
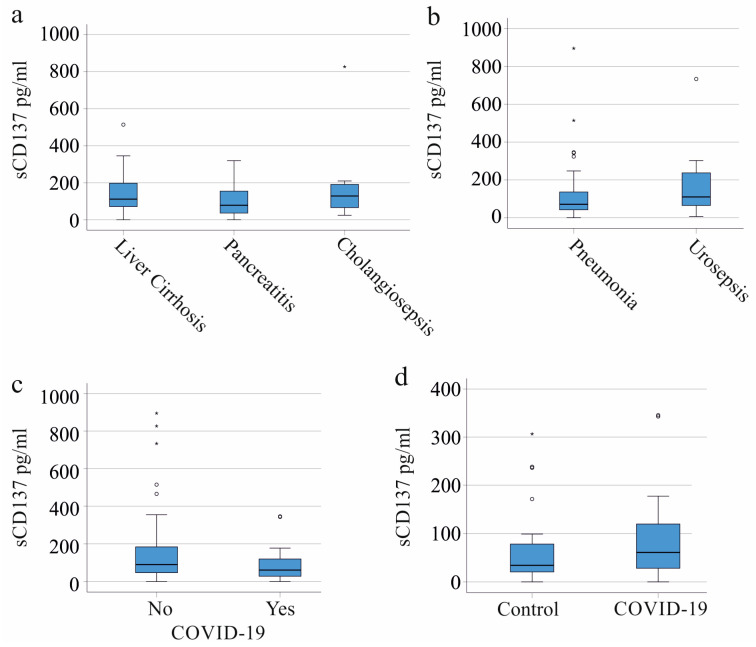
The sCD137 in the plasma of critically ill patients stratified for underlying diseases and causes of severe illness. (**a**) Plasma sCD137 levels of the SIRS/sepsis patients with different underlying diseases; (**b**) Plasma sCD137 levels of the SIRS/sepsis patients with pneumonia and urosepsis; (**c**) Plasma sCD137 levels of the SIRS/sepsis patients without and with SARS-CoV-2 infection; (**d**) Plasma sCD137 levels of the SIRS/sepsis patients with SARS-CoV-2 infection and the healthy controls. Small circles and asterisks mark outliers.

Underlying diseases may affect the levels of biomarkers in sepsis, and, e.g., CRP is low in patients with liver cirrhosis (Table 1). In our cohort, 30 patients had underlying liver cirrhosis. Of the others, 31 developed SIRS/sepsis from pancreatitis and 9 from cholangitis. Plasma sCD137 levels showed no difference between these groups (Figure 2a). Additional major causes of severe illness in our SIRS/sepsis cohort were pulmonary infections (52 patients) and urinary tract infections (14 patients), with comparable plasma sCD137 levels (Figure 2b).

Twenty-three patients in our SIRS/sepsis cohort were infected with SARS-CoV-2. Their leukocyte counts and CRP levels were similar to the entire cohort, but they had lower procalcitonin levels (Table 1). Plasma sCD137 levels in severely ill patients, both with and without COVID-19, were similar (Figure 2c). Notably, SIRS/sepsis patients with a SARS-CoV-2 infection had sCD137 levels akin to the 22 healthy controls described in Table 1 (*p* = 0.159) (Figure 2d).

### 2.3. Plasma sCD137 Levels in Relation to Interventions and Vasopressor Therapy

Plasma sCD137 levels in our SIRS/sepsis cohort were similar between the 52 patients on dialysis and those without extracorporeal organ support (Table 2). Mechanical ventilation in 94 patients and vasopressor therapy in 92 patients did not alter the plasma sCD137 levels (Table 2).

### 2.4. Plasma sCD137 and Inflammation Markers

In our SIRS/sepsis cohort, the plasma sCD137 positively correlated with procalcitonin (r = 0.186, *p* = 0.024) and IL-6 (r = 0.181, *p* = 0.029), and negatively with CRP (r = −0.187, *p* = 0.022). The correlations with CRP (r = −0.160, *p* = 0.079) and procalcitonin (r = 0.153, *p* = 0.099) were not significant when patients with liver cirrhosis were excluded, whereas IL-6 still positively correlated with sCD137 (r = 0.202, *p* = 0.029).

IL-6 positively correlated with CRP in our SIRS/sepsis cohort (r = 0.230, *p* = 0.006), and this association was still significant when the patients with liver cirrhosis were excluded (r = 0.298, *p* = 0.001).

The leukocyte count was not associated with the plasma sCD137 levels (r = 0.066, *p* = 0.422). Neutrophils (r = 0.095, *p* = 0.253), basophils (r = 0.030, *p* = 0.717), eosinophils (r = −0.096, *p* = 0.247), monocytes (r = 0.021, *p* = 0.801), lymphocytes (r = −0.089, *p* = 0.288), and immature granulocytes (r = 0.079, *p* = 0.348) did not correlate with sCD137.

In the COVID-19 patients, the plasma sCD137 did not correlate with CRP (r = −0.231, *p* = 0.289), procalcitonin (r = 0.129, *p* = 0.559), ferritin (r = 0.208, *p* = 0.341), or IL-6 (r = −0.122, *p* = 0.600). The leukocyte count (r = −0.201, *p* = 0.358), neutrophils (r = −0.078, *p* = 0.730), basophils (r = −0.076, *p* = 0.736), eosinophils (r = −0.164, *p* = 0.465), monocytes (r = −0.120, *p* = 0.595), lymphocytes (r = −0.399, *p* = 0.066), and immature granulocytes (r = 0.080, *p* = 0.724) did not correlate with sCD137.

### 2.5. Plasma sCD137 in Gram-Negative and Gram-Positive Bacterial Infections

**Figure 3:**
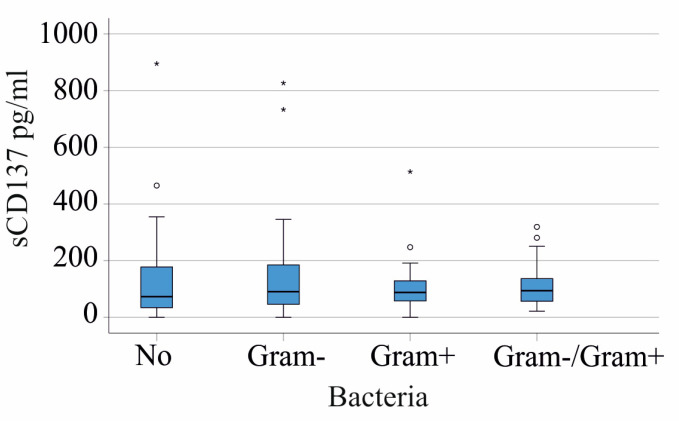
The sCD137 in the plasma of SIRS/sepsis patients according to bacterial infection. Plasma sCD137 levels of the SIRS/sepsis patients with no bacteria detected (No), infected with Gram-negative bacteria (Gram−), infected with Gram+ bacteria (Gram+), or both types of bacteria. Small circles and asterisks mark outliers.

In our SIRS/sepsis cohort, the plasma sCD137 levels in the 53 patients without detectable infectious agents in the blood were comparable to those in the 59 patients infected with Gram-negative bacteria and the 21 patients with Gram-positive bacterial infections. The 20 patients coinfected with both Gram-negative and Gram-positive bacteria had sCD137 levels similar to noninfected patients (Figure 3).

### 2.6. Plasma sCD137 and Survival

**Figure 4:**
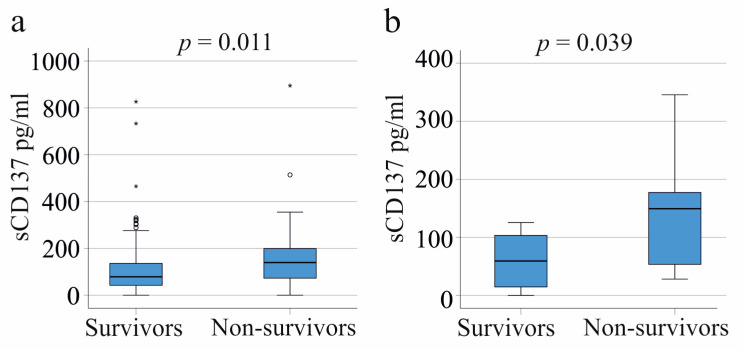
The sCD137 in the plasma of critically ill patients according to survival. (**a**) Plasma sCD137 levels of survivors and non-survivors; (**b**) Plasma sCD137 levels of COVID-19 survivors and non-survivors. Small circles and asterisks mark outliers.

Among the 153 SIRS/sepsis patients, the plasma sCD137 levels at the time of intensive care unit admission were higher in the 36 non-survivors compared to the survivors (Figure 4a). Similarly, in the COVID-19 patient group, higher sCD137 levels in plasma were observed in the nine non-survivors (Figure 4b).

The ROC curve for the prediction of non-survival in the whole SIRS/sepsis cohort showed an area under the curve (AUC) of 0.643 for sCD137 (sensitivity 54%, specificity 74%, cut-off 131.6 pg/mL) and an AUC of 0.758 in the COVID-19 cohort (sensitivity 56%, specificity 100%, cut-off 137.5 pg/mL). According to this analysis, plasma sCD137 is not well-suited for the prediction of death.

## 3. Discussion

Our analysis revealed that severely ill patients had higher plasma sCD137 levels compared to healthy controls. Additionally, non-surviving SIRS/sepsis patients exhibited even higher sCD137 levels. Given that sCD137 levels indicate T cell activation [5], these findings suggest a significant T cell involvement in SIRS/sepsis. Recognizing this could provide key insights into the disease mechanism and open up novel potential therapeutic strategies.

There is some evidence for higher circulating sCD137 levels in patients with different inflammatory diseases [5,22,24]. The sCD137 serum levels of patients with rheumatoid arthritis were greatly increased and were about 20-fold higher in contrast to healthy controls [5]. The serum concentrations of sCD137 of patients with acute pancreatitis were also elevated in contrast to healthy controls, and were related to disease severity and mortality [24]. The higher sCD137 of patients with HCV-related liver cirrhosis correlated with serum tumor necrosis factor levels [18].

To our knowledge, the elevated serum levels of sCD137 in sepsis patients with various etiologies, compared to healthy controls, have not been previously described. Only one study on severe COVID-19-induced viral sepsis noted higher sCD137 compared to healthy controls [22]. The increased sCD137 in this group might result from T cell responses to the viral infection or the severity of the illness itself. In our SIRS/sepsis cohort, SARS-CoV-2 infection did not correlate with higher sCD137 levels, implying the rise is more tied to critical illness than specifically to COVID-19. The referenced study, which involved 62 severe COVID-19 patients and 16 healthy controls [22], had a larger COVID-19 sample size than ours. This might account for the observed differences between our findings and theirs [22].

The ROC curve showed a cut-off value of 49.43 pg/mL sCD137 for the diagnosis of sepsis, with a sensitivity of 72% and a specificity of 73%. The diagnostic performance of the sCD137 analysis for the sepsis diagnosis is comparable to the diagnostic accuracy of CRP and procalcitonin [27]. Studies in larger cohorts have to prove whether the analysis of sCD137 in addition to CRP and procalcitonin adds a further diagnostic value.

Sepsis patients usually have increased neutrophils and monocytes, and lower lymphocytes, in comparison to the normal reference values [28]. Plasma sCD137 levels did not correlate with any of the analyzed immune cell populations in blood, and from our study, there is no evidence that sCD137 affects the white blood cell count or composition in sepsis. Previous studies showed that sCD137 regulates the activation status of immune cells [5] and the characterization of subpopulations, such as Th1 and Th2 T cells, may identify the associations of sCD137 with immune cell function in sepsis.

In our study, the plasma sCD137 levels in the SIRS/sepsis patients were not associated with disease severity; patients with SIRS, sepsis, and septic shock showed similar levels. Additionally, the sCD137 levels were not elevated in patients requiring dialysis, ventilation, or vasopressor treatment.

Furthermore, the sCD137 levels were not linked to the underlying disease. While patients with liver cirrhosis typically exhibit increased serum sCD137 [18], our septic patients with liver cirrhosis only showed a trend towards higher plasma sCD137 levels. This difference was not significant compared to septic patients without liver cirrhosis. The limited number of patients with liver cirrhosis in our cohort might explain this observation. If sCD137 is to be developed as a new biomarker for infections in patients with liver cirrhosis, larger studies are needed. Notably, to our knowledge, sCD137 has not been previously analyzed in septic patients with liver cirrhosis.

While previous studies linked pancreatitis to elevated serum sCD137 levels [24], we did not observe this difference in our SIRS/sepsis cohort. This indicates that severe illness, more so than underlying conditions like pancreatitis, influences serum sCD137 levels in SIRS/sepsis patients. Therefore, our data offer new perspectives on the factors affecting sCD137 levels.

Prior research has shown a positive correlation between sCD137 and CRP. For instance, in patients with acute coronary syndromes, serum sCD137 was positively linked with CRP [29]. Similarly, a positive correlation was observed in patients with acute ST-segment elevation myocardial infarction [30]. Contrarily, in our SIRS/sepsis cohort, sCD137 was negatively associated with serum CRP levels. Given that sCD137 is an inhibitory molecule indicative of T cell involvement [5], the exact role of elevated sCD137 in reducing CRP warrants further investigation.

IL-6 is a key factor in the regulation of acute-phase proteins, such as CRP [31], and was positively correlated with CRP and sCD137 in our SIRS/sepsis cohort. The negative association of sCD137 and CRP, and the positive correlation of sCD137 with IL-6, cannot be explained currently and requires further study. It is possible that the negative correlation of sCD137 with CRP is related to the slightly higher levels of sCD137 and the reduced levels of CRP in liver cirrhosis [32], and the correlation of CRP and sCD137 was not significant after the exclusion of patients with liver cirrhosis. The loss of this correlation could also be related to the smaller size of the cohort, and this has to be further evaluated in larger study groups.

CD137 can activate different cells and induce the expression of IL-6 by these cells [6], and the positive association of IL-6 and sCD137 indicates that the immunosuppressive effects of sCD137 are less effective in SIRS/sepsis patients. CD137 agonists have been shown to ameliorate autoimmune diseases [5] and may also have a role in SIRS/sepsis therapy. Present observations suggest that CD137 agonists, beside recombinant sCD137, may become a preferred treatment approach.

Procalcitonin serves as a biomarker for bacterial infections [33]. While plasma sCD137 correlated positively with procalcitonin levels, it did not increase in the plasma of patients with bacterial infections compared to noninfected patients. This observation might be attributed to potential false-negative microbiological results.

Our data indicate that bacterial infection does not influence plasma sCD137 levels in SIRS/sepsis patients. Previous studies demonstrated that CD137 has protective effects in Gram-positive infections, but can be harmful in mice infected with both Gram-negative and Gram-positive bacteria, or when injected with lipopolysaccharides [10,11,12]. Interestingly, during the immunosuppressive phase of severe infections, administering CD137-Fc enhanced the recovery from Gram-negative *Acinetobacter pneumonia* [13]. These findings in animal studies highlight the dual role of CD137 in various stages of infectious diseases [10,11,12]. Monitoring sCD137 levels throughout sepsis could shed light on the impact of CD137 signaling in bacterial infections.

Our current analysis did not observe variations in sCD137 plasma levels based on the type of bacterial infection. This observation suggests that elevated plasma sCD137 might be more indicative of the severity of illness rather than the specific infectious agent.

Plasma sCD137 has been identified as a suitable marker to monitor CD137 activity, especially when prompted by CD137 targets; this is because CD137 costimulation results in the induction of both CD137 and sCD137 expressions and their subsequent circulation [29]. Notably, lymphocyte activation correlates with an increase in sCD137 levels approximately 3 days afterward, hinting at the potential role of sCD137 in dampening T cell activation and CD137-dependent costimulatory signaling pathways [14]. Given that plasma sCD137 signifies prior CD137 activation [34], its elevated levels in SIRS/sepsis patients implies an early activation of CD137 signaling during severe illness.

In our SIRS/sepsis cohort, non-survivors exhibited elevated plasma sCD137 levels. A similar increase in plasma sCD137 was observed in non-surviving COVID-19 patients. Previous studies have identified associations between elevated sCD137 and mortality in metastatic cancer patients, where high systemic sCD137 correlated with reduced progression-free and overall survival [35]. Elevated serum sCD137 levels have also been linked to mortality in patients with acute pancreatitis [24] and to sudden death in those with acute coronary syndrome [36]. This observation is, however, not of diagnostic value, because the sensitivity and specificity of sCD137 for the prediction of death was low.

When analyzing biomarkers, it is essential to identify potential confounding factors, like age or gender. Consistent with prior studies [24,37], plasma sCD137 levels were similar between males and females. While age showed a weak positive correlation with plasma sCD137 in the control group, but not in the patient cohort, similar associations between sCD137 and age were observed in patients with nonsmall-cell lung cancer [37].

This study has some limitations. CD137 was shown to polarize T helper cells toward Th1 cells [5], but we did not analyze the different T cell populations of the patients. Additionally, the subcohort of patients with SARS-CoV-2 infection was small, which could limit the statistical power.

## 4. Materials and Methods

### 4.1. Patients

Between August 2018 and January 2023, plasma was collected from 153 patients at the University Hospital of Regensburg. Based on the Sepsis-3 criteria, 37 patients had sepsis and 77 had septic shock [38]. The remaining 39 patients, initially suspected of sepsis but who did not develop it during follow-up, met the systemic inflammatory response syndrome (SIRS) criteria [39]. They had a sepsis-related organ failure assessment (SOFA) score of less than 2 and, per the Sepsis-3 criteria, did not have sepsis [38,39]. Patients with multiresistant infections, or those infected with HIV or viral hepatitis, were excluded. All patients were admitted to the intensive care unit.

Control participants included 11 healthy females and 11 males, with no significant difference in sex distribution compared to patients (*p* = 0.059). The control group’s age range was 58 (40–67) years, statistically similar to the patient group (*p* = 0.449).

### 4.2. Analysis of sCD137 and Interleukin-6

Blood samples were obtained between 12 and 24 h after the admission of the patients to the intensive care unit. EDTA was the anticoagulant, and plasma was prepared. The DuoSet ELISA used to measure the human sCD137 in plasma was from R&D Systems (Wiesbaden, Nordenstadt, Germany), and the ELISA was performed as recommended by the distributor. Plasma was diluted 1:2-fold for analysis. The IL-6 DuoSet ELISA kit from R&D Systems was used for the IL-6 analysis; therefore, the plasma was also two-fold diluted.

### 4.3. Statistical Analysis

All figures display the data as boxplots, showing the minimum, maximum, median, and both the first and third quartiles. Outliers are indicated by circles (sCD137 levels > 1.5 × the interquartile range from either quartile) and asterisks (sCD137 levels >3.0 × the interquartile range from either quartile). Table data present the median, minimum, and maximum values. Statistical analyses were conducted using the Chi-Square test, Mann–Whitney U test, Kruskal–Wallis test, and Spearman’s correlation in IBM SPSS Statistics 26.0. A *p*-value < 0.05 was deemed significant.

## 5. Conclusions

Our analysis indicates that elevated plasma sCD137 levels correlate with mortality in SIRS/sepsis patients. While the plasma sCD137 levels in the septic patients with COVID-19 resembled those of the healthy controls, non-survivors displayed higher levels. Septic patients with liver cirrhosis exhibited elevated sCD137, albeit not statistically significant. This warrants further investigation in larger studies. The plasma sCD137 levels in the SIRS/sepsis patients are not influenced by the underlying causes of SIRS/sepsis, nor by bacterial or SARS-CoV-2 infections, hinting at a link to immune dysfunction in severe illness.

## Figures and Tables

**Table 1:** Characteristics of the whole cohort, the subgroup of patients with COVID-19, the subgroup of patients with liver cirrhosis, and the 22 healthy controls. IL-6 was not measured in the plasma of all patients because of the lack of plasma, and the superscript numbers indicate for how many patients IL-6 has been analyzed. The respective *p*-values are * *p* < 0.05 and ** *p* < 0.01 for the comparison of the whole cohort and the subcohort of patients with COVID-19; ^§§§^ *p* < 0.001 for the comparison of the whole cohort and the subcohort of patients with liver cirrhosis; ^%%%^ *p* < 0.001 for the comparison of the whole cohort and the controls.

Parameters	Whole Cohort	COVID-19 Patients	Cirrhosis Patients	Controls
Males/Females	106/47	15/8	21/9	11/11
Age (years)	59 (21–93)	62 (29–80)	57 (31–75)	58 (40–67)
C-reactive Protein mg/L	156 (12–697) ^§§§^	129 (44–472)	83 (12–236) ^§§§^	not determined
Procalcitonin ng/mL	1.16 (0.05–270.00) *	0.57 (0.08–65.40) *	1.20 (0.10–65.18)	not determined
SIRS/Sepsis/Septic Shock	39/37/77 **	0/3/20 **	10/6/14	not determined
IL-6 pg/mL	88.9 (0–5701.7) ^145%%%^	48.6 (5.6–1810.2) ^21%%%^	129.4 (14.05–2546.6) ^21^	6.5 (0–48.3) ^21%%%^
Ferritin ng/mL	not determined	970 (200–17,846)	not determined	not determined
Leukocytes n × 10^9^/L	10.24 (0.06–1586.00)	8.64 (2.78–18.47)	10.32 (2.51–1586.00)	not determined
Neutrophils n/nL	7.69 (0.01–70.20)	6.83 (0.14–48.40)	7.85 (1.46–25.25)	not determined
Basophils n/nL	0.04 (0–0.90)	0.03 (0–0.60)	0.04 (0–0.42)	not determined
Eosinophils n/nL	0.13 (0–8.80)	0.04 (0–8.80)	0.14 (0.01–2.89)	not determined
Monocytes n/nL	0.79 (0–45.00)	0.64 (0–10.90)	1.00 (0.41–3.29)	not determined
Lymphocytes n/nL	0.96 (0.08 –28.60)	0.70 (0.16 –28.60)	0.79 (0.16–5.74)	not determined
Immature Granulocytes n/nL	0.12 (0–6.19)	0.24 (0–3.84)	0.11 (0.01–1.18)	not determined

**Table 2:** Plasma sCD137 levels of patients on dialysis, ventilation, or vasopressor therapy in comparison to patients without these interventions/therapies.

Intervention/Drug	No	Yes	*p*-Value
Dialysis	77.6 (0–826.5) pg/mL	113.5 (0–895.1) pg/mL	0.100
Ventilation	86.6 (0–733.2) pg/mL	89.4 (0–895.1) pg/mL	0.855
Catecholamine	78.4 (0–733.2) pg/mL	89.4 (0–895.1) pg/mL	0.873

## Data Availability

Data supporting reported results can be obtained from the corresponding author.
